# *catena*-Poly[[(dimethyl­formamide-κ*O*)cobalt(II)]-bis­[μ-(4-nitro­phenyl)­cyanamido]-κ^2^ *N* ^1^:*N* ^3^;κ^2^ *N* ^3^:*N* ^1^]

**DOI:** 10.1107/S160053681000557X

**Published:** 2010-02-24

**Authors:** Hossein Chiniforoshan, Bahare Shirinfar, Soghra Jalilpour, Hamid Reza Khavasi

**Affiliations:** a Department of Chemistry, Isfahan University of Technology, Isfahan 84456-38111, Iran; b Department of Chemistry, Shahid Beheshti University, G. C., Evin, Tehran 1983963113, Iran

## Abstract

In the title coordination polymer, [Co(C_7_H_4_N_3_O_2_)_2_(C_3_H_7_NO)]_*n*_, the Co^II^ atom is five-coordinated in a distorted square-pyramidal CoON_4_ geometry with the O atom from a dimethyl­formamide mol­ecule in an equatorial position. The bridging phenyl­cyanamide anions generate an infinite chain propagating in [001].

## Related literature

For background to models of ligand bonding, see: Storhoff & Lewis (1977 ▶); Chisholm *et al.* (1987 ▶); Crutchley *et al.* (1999 ▶). For related structures, see: Escuer *et al.* (2003*a* ▶,*b* ▶, 2004 ▶); Chiniforoshan *et al.* (2009 ▶). For further synthetic details, see: Crutchley & Naklicki (1989 ▶).
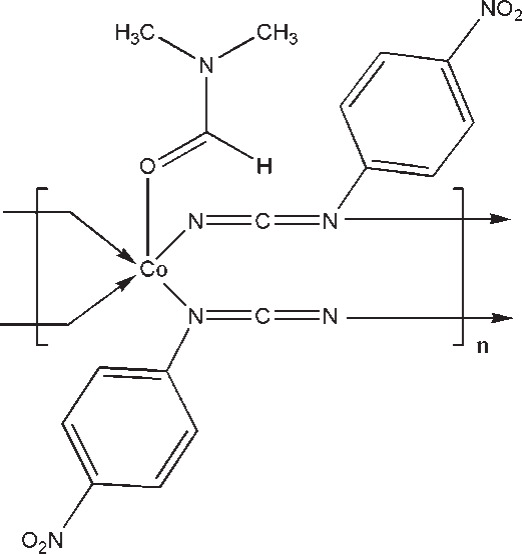


## Experimental

### 

#### Crystal data

[Co(C_7_H_4_N_3_O_2_)_2_(C_3_H_7_NO)]
*M* *_r_* = 456.29
Monoclinic, 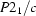

*a* = 21.8692 (16) Å
*b* = 8.8517 (6) Å
*c* = 9.9827 (8) Å
β = 100.151 (6)°
*V* = 1902.2 (2) Å^3^
*Z* = 4
Mo *K*α radiation
μ = 0.95 mm^−1^
*T* = 120 K
0.30 × 0.12 × 0.10 mm

#### Data collection

STOE IPDS II diffractometer
Absorption correction: numerical [optical; *X-RED* and *X-SHAPE* (Stoe & Cie, 2005 ▶)] *T*_min_ = 0.740, *T*_max_ = 0.800
22258 measured reflections
5131 independent reflections
4161 reflections with *I* > 2σ(*I*)
*R*_int_ = 0.074

#### Refinement

*R*[*F* ^2^ > 2σ(*F* ^2^)] = 0.052
*wR*(*F* ^2^) = 0.104
*S* = 1.20
5131 reflections
273 parameters
H-atom parameters constrained
Δρ_max_ = 0.35 e Å^−3^
Δρ_min_ = −0.70 e Å^−3^

### 

Data collection: *X-AREA* (Stoe & Cie, 2005 ▶); cell refinement: *X-AREA*; data reduction: *X-AREA*; program(s) used to solve structure: *SHELXS97* (Sheldrick, 2008 ▶); program(s) used to refine structure: *SHELXL97* (Sheldrick, 2008 ▶); molecular graphics: *ORTEP-3* (Farrugia, 1997 ▶); software used to prepare material for publication: *WinGX* (Farrugia, 1999 ▶).

## Supplementary Material

Crystal structure: contains datablocks I, global. DOI: 10.1107/S160053681000557X/hb5302sup1.cif

Structure factors: contains datablocks I. DOI: 10.1107/S160053681000557X/hb5302Isup2.hkl

Additional supplementary materials:  crystallographic information; 3D view; checkCIF report

## Figures and Tables

**Table 1:** Selected bond lengths (Å)

Co1—O5	1.9807 (19)
Co1—N3	1.994 (2)
Co1—N5	2.198 (2)
Co1—N6^i^	1.971 (2)
Co1—N2^i^	2.2896 (19)

Symmetry code: (i) 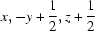.

**Fig. 1.**
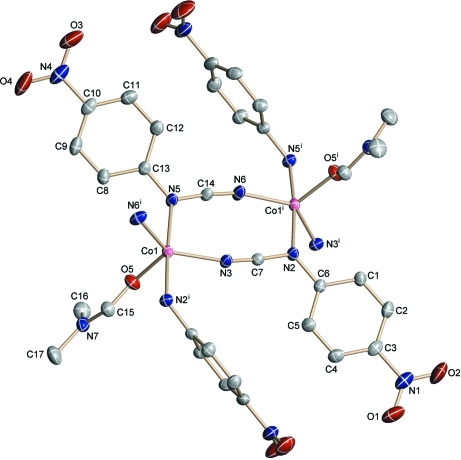
View of (I) (30% probability displacement ellipsoids), (i) x,-y+1/2,z-1/2.

## supplementary crystallographic information

### Comment

Phenylcyanamide ligands (Pcyd) are interesting and practically ligands from the synthetic and magnetic point of view. Recently, we have reported the first magnetic measurements on systems with different dimensionality containing the MnII-(NCN)2- unit (Escuer *et al.*, 2003a,b) the cyanamido group(NCN) being coordinated in the end-to-end mode. From the synthetic point of view, pcyd ligands offer a wide range of possibilities based on the use different X-pcyd derivatives which can coordinate properties of the two nitrogen atoms: one *N*-nitrile, which coordinate preferently and one *N*-amide atom, with characteristic bond parameters in each case (Escuer *et al.*, 2004). Following our work with this family of ligands, we now report four new Co^II^-Xpcyd compounds (using 4-nitro,4-fluoro,4-chloro,4-bromophenylcyanamide) in combination with the dimethylformamide ligand. These compounds contain the unusual end-to-end phenyl-cyanamide bridge and give supramolecular one dimensional network by means of H-bonds involving the N-amid atoms of the phenylcyanamide ligands. Side-on coordination of a nitrile group is extremely rare (Storhoff & Lewis, 1977) but is more common for cyanamide ligands due to the participation of the nitrile lone pair in bridging interaction (Chisholm *et al.*, 1987). We are attempting to construct conductive polymer chains that are cross-linked by cyanamide groups to a coordination complex. Conductivity within this linked system will arise provided the polymer *p*-pi orbitals and the metal dp orbital are both symmetry and energy matched (Crutchley *et al.*, 1999). More recently various aromatic cyanamide complexes have been studied by x-ray crystallography (Chiniforoshan *et al.*, 2009).

We report here the synthesis and crystal structure of the title complex, (I).

In the molecule of the title compound, (I), (Fig. 1) the selected bond lengths and angles are listed in Table 1. in this molecule, the {Co(4—NO_2_-pcyd)_2_(DMF)}_n_ one-dimensional chain coordination polymer bridged by 4-NO_2_-phenylcyanamide. Each cobalt atom has a distored square pyramidal geometry, that nitrogen atoms are in equatirial position and oxygen atom from DMF molecules is in axial position, Table 1. The dihedral angle between adjacent phenyl rings in the polymeric chain is 89.02 (10) °.

### Experimental

4-Nitrophenylcyanamide (Crutchley *et al.*,1989) (0.326 g, 0.5 mmol) was dissolved in methanol (25 ml) and was added slowly to a solution of cobalt(II) acetate (0.249 g, 0.25 mmol) in methanol (25 ml). The mixture was stirred for 5 h. The resulting solid was filtered off and violet needles of (I) obtained by dissolving in DMF then diffused by acetonitrile after 2 week.

### Refinement

All of the H atoms were positioned geometrically, with C—H = 0.93 Å for aromatic and aldehyde H atoms and with C—H = 0.93 Å for methyl hydrogens, and constrained to ride on their parent atoms, with U_iso_(H) = 1.2U_eq_(C).

### Crystal data

**Table:**

[Co(C_7_H_4_N_3_O_2_)_2_(C_3_H_7_NO)]	*F*(000) = 932
*M**_r_* = 456.29	*D*_x_ = 1.593 Mg m^−^^3^
Monoclinic, *P*2_1_/*c*	Mo *K*α radiation, λ = 0.71073 Å
Hall symbol: -P 2ybc	Cell parameters from 987 reflections
*a* = 21.8692 (16) Å	θ = 1.9–29.3°
*b* = 8.8517 (6) Å	µ = 0.95 mm^−^^1^
*c* = 9.9827 (8) Å	*T* = 120 K
β = 100.151 (6)°	Needle, violet
*V* = 1902.2 (2) Å^3^	0.3 × 0.12 × 0.1 mm
*Z* = 4	

### Data collection

**Table:**

STOE IPDS II diffractometer	5131 independent reflections
Radiation source: fine-focus sealed tube	4161 reflections with *I* > 2σ(*I*)
graphite	*R*_int_ = 0.074
rotation method scans	θ_max_ = 29.3°, θ_min_ = 1.9°
Absorption correction: numerical [optical; *X-RED* and *X-SHAPE* (Stoe & Cie, 2005)]	*h* = −30→30
*T*_min_ = 0.740, *T*_max_ = 0.800	*k* = −12→12
22258 measured reflections	*l* = −13→13

### Refinement

**Table:**

Refinement on *F*^2^	Primary atom site location: structure-invariant direct methods
Least-squares matrix: full	Secondary atom site location: difference Fourier map
*R*[*F*^2^ > 2σ(*F*^2^)] = 0.052	Hydrogen site location: inferred from neighbouring sites
*wR*(*F*^2^) = 0.104	H-atom parameters constrained
*S* = 1.20	*w* = 1/[σ^2^(*F*_o_^2^) + (0.0287*P*)^2^ + 1.1087*P*] where *P* = (*F*_o_^2^ + 2*F*_c_^2^)/3
5131 reflections	(Δ/σ)_max_ = 0.023
273 parameters	Δρ_max_ = 0.35 e Å^−^^3^
0 restraints	Δρ_min_ = −0.69 e Å^−^^3^

### Special details

**Table:**

Geometry. All esds (except the esd in the dihedral angle between two l.s. planes) are estimated using the full covariance matrix. The cell esds are taken into account individually in the estimation of esds in distances, angles and torsion angles; correlations between esds in cell parameters are only used when they are defined by crystal symmetry. An approximate (isotropic) treatment of cell esds is used for estimating esds involving l.s. planes.
Refinement. Refinement of *F*^2^ against ALL reflections. The weighted *R*-factor wR and goodness of fit *S* are based on *F*^2^, conventional *R*-factors *R* are based on *F*, with *F* set to zero for negative *F*^2^. The threshold expression of *F*^2^ > σ(*F*^2^) is used only for calculating *R*-factors(gt) etc. and is not relevant to the choice of reflections for refinement. *R*-factors based on *F*^2^ are statistically about twice as large as those based on *F*, and *R*- factors based on ALL data will be even larger.

### Fractional atomic coordinates and isotropic or equivalent isotropic displacement parameters (Å^2^)

**Table:**

	*x*	*y*	*z*	*U*_iso_*/*U*_eq_	
C1	0.40669 (12)	0.4544 (3)	0.2890 (2)	0.0367 (5)	
H1	0.3765	0.5107	0.2332	0.044*	
C2	0.46736 (13)	0.4600 (3)	0.2693 (3)	0.0418 (6)	
H2	0.4782	0.5200	0.2007	0.050*	
C3	0.51209 (12)	0.3757 (3)	0.3524 (3)	0.0411 (6)	
C4	0.49727 (12)	0.2877 (3)	0.4559 (3)	0.0432 (6)	
H4	0.5279	0.2326	0.5116	0.052*	
C5	0.43659 (12)	0.2821 (3)	0.4759 (2)	0.0392 (6)	
H5	0.4264	0.2228	0.5457	0.047*	
C6	0.39001 (10)	0.3643 (3)	0.3927 (2)	0.0299 (4)	
C7	0.31504 (10)	0.2978 (3)	0.5177 (2)	0.0295 (4)	
C8	0.08686 (12)	0.0534 (3)	0.6636 (2)	0.0391 (6)	
H8	0.1158	−0.0163	0.7060	0.047*	
C9	0.02663 (13)	0.0479 (4)	0.6859 (3)	0.0455 (7)	
H9	0.0145	−0.0259	0.7420	0.055*	
C10	−0.01572 (12)	0.1535 (4)	0.6238 (3)	0.0457 (7)	
C11	0.00043 (13)	0.2613 (4)	0.5372 (3)	0.0479 (7)	
H11	−0.0289	0.3300	0.4948	0.057*	
C12	0.06063 (13)	0.2663 (3)	0.5141 (3)	0.0421 (6)	
H12	0.0720	0.3388	0.4557	0.051*	
C13	0.10495 (11)	0.1630 (3)	0.5777 (2)	0.0320 (5)	
C14	0.17962 (11)	0.2310 (3)	0.4517 (2)	0.0329 (5)	
C15	0.26634 (13)	−0.1792 (3)	0.7963 (3)	0.0404 (6)	
H15	0.2886	−0.1460	0.8791	0.049*	
C16	0.22430 (18)	−0.3852 (4)	0.6534 (3)	0.0590 (8)	
H16A	0.2522	−0.4419	0.6084	0.071*	
H16B	0.2065	−0.3041	0.5956	0.071*	
H16C	0.1918	−0.4503	0.6726	0.071*	
C17	0.28133 (19)	−0.4310 (4)	0.8862 (4)	0.0623 (9)	
H17A	0.2471	−0.4804	0.9163	0.075*	
H17B	0.3056	−0.3781	0.9612	0.075*	
H17C	0.3068	−0.5050	0.8522	0.075*	
Co1	0.246533 (14)	0.14018 (3)	0.72734 (3)	0.02718 (9)	
N1	0.57585 (12)	0.3801 (4)	0.3298 (3)	0.0590 (7)	
N2	0.32762 (9)	0.3587 (2)	0.40707 (17)	0.0315 (4)	
N3	0.29935 (9)	0.2463 (2)	0.61327 (19)	0.0341 (4)	
N4	−0.07929 (13)	0.1482 (4)	0.6502 (3)	0.0655 (8)	
N5	0.16710 (9)	0.1668 (2)	0.56040 (18)	0.0331 (4)	
N6	0.19618 (10)	0.2829 (3)	0.3580 (2)	0.0429 (5)	
N7	0.25790 (11)	−0.3243 (2)	0.7787 (2)	0.0417 (5)	
O1	0.61519 (11)	0.3055 (4)	0.4033 (3)	0.0845 (9)	
O2	0.58758 (13)	0.4571 (5)	0.2362 (4)	0.1081 (12)	
O3	−0.11534 (13)	0.2481 (5)	0.6018 (3)	0.0965 (11)	
O4	−0.09355 (14)	0.0446 (5)	0.7210 (3)	0.1003 (11)	
O5	0.24632 (10)	−0.0826 (2)	0.70889 (18)	0.0443 (4)	

### Atomic displacement parameters (Å^2^)

**Table:**

	*U*^11^	*U*^22^	*U*^33^	*U*^12^	*U*^13^	*U*^23^
C1	0.0355 (12)	0.0426 (14)	0.0319 (12)	−0.0053 (11)	0.0057 (9)	0.0042 (10)
C2	0.0403 (14)	0.0475 (15)	0.0397 (14)	−0.0138 (12)	0.0128 (11)	−0.0023 (11)
C3	0.0308 (12)	0.0495 (15)	0.0439 (14)	−0.0093 (11)	0.0090 (10)	−0.0164 (12)
C4	0.0332 (13)	0.0552 (17)	0.0398 (14)	0.0041 (12)	0.0024 (10)	−0.0030 (12)
C5	0.0384 (13)	0.0484 (15)	0.0308 (12)	0.0026 (11)	0.0063 (10)	0.0051 (11)
C6	0.0299 (10)	0.0337 (11)	0.0258 (10)	−0.0057 (10)	0.0040 (8)	−0.0036 (9)
C7	0.0275 (10)	0.0345 (11)	0.0253 (10)	−0.0014 (9)	0.0014 (8)	−0.0020 (9)
C8	0.0400 (14)	0.0454 (15)	0.0327 (12)	−0.0077 (11)	0.0083 (10)	0.0024 (10)
C9	0.0438 (15)	0.0599 (18)	0.0349 (13)	−0.0200 (13)	0.0126 (11)	−0.0042 (12)
C10	0.0331 (12)	0.071 (2)	0.0344 (12)	−0.0112 (13)	0.0104 (10)	−0.0166 (13)
C11	0.0357 (14)	0.067 (2)	0.0403 (14)	0.0050 (13)	0.0050 (11)	−0.0051 (13)
C12	0.0382 (14)	0.0554 (17)	0.0331 (13)	−0.0019 (12)	0.0073 (10)	0.0057 (11)
C13	0.0306 (11)	0.0429 (14)	0.0223 (10)	−0.0048 (10)	0.0038 (8)	−0.0019 (9)
C14	0.0255 (11)	0.0475 (14)	0.0250 (11)	−0.0020 (10)	0.0021 (8)	0.0006 (9)
C15	0.0488 (15)	0.0371 (13)	0.0353 (13)	−0.0011 (11)	0.0070 (11)	−0.0001 (9)
C16	0.068 (2)	0.0443 (17)	0.065 (2)	−0.0063 (15)	0.0124 (16)	−0.0074 (14)
C17	0.081 (3)	0.0446 (17)	0.063 (2)	−0.0005 (17)	0.0191 (18)	0.0159 (15)
Co1	0.03239 (15)	0.02822 (15)	0.02268 (13)	0.00103 (14)	0.00971 (10)	0.00032 (12)
N1	0.0367 (13)	0.077 (2)	0.0663 (17)	−0.0085 (13)	0.0166 (12)	−0.0189 (15)
N2	0.0314 (9)	0.0402 (10)	0.0232 (8)	−0.0030 (9)	0.0053 (7)	0.0037 (8)
N3	0.0279 (10)	0.0477 (12)	0.0263 (9)	−0.0028 (8)	0.0037 (7)	0.0051 (8)
N4	0.0382 (14)	0.108 (3)	0.0527 (15)	−0.0140 (17)	0.0163 (12)	−0.0224 (17)
N5	0.0303 (9)	0.0470 (12)	0.0226 (9)	−0.0016 (8)	0.0062 (7)	0.0051 (8)
N6	0.0295 (10)	0.0722 (16)	0.0268 (10)	−0.0046 (10)	0.0046 (8)	0.0128 (10)
N7	0.0458 (12)	0.0328 (11)	0.0499 (12)	−0.0012 (10)	0.0180 (10)	0.0008 (9)
O1	0.0370 (12)	0.122 (3)	0.094 (2)	0.0105 (15)	0.0106 (13)	−0.0062 (18)
O2	0.0537 (17)	0.154 (3)	0.128 (3)	−0.0070 (18)	0.0467 (18)	0.039 (2)
O3	0.0409 (14)	0.154 (3)	0.097 (2)	0.0129 (17)	0.0192 (14)	−0.003 (2)
O4	0.0613 (17)	0.146 (3)	0.105 (2)	−0.0246 (19)	0.0463 (16)	0.010 (2)
O5	0.0588 (12)	0.0305 (8)	0.0408 (10)	0.0021 (8)	0.0012 (9)	0.0004 (7)

### Geometric parameters (Å, °)

**Table:**

C1—C2	1.376 (4)	C13—N5	1.400 (3)
C1—C6	1.405 (3)	C14—N6	1.157 (3)
C1—H1	0.9300	C14—N5	1.296 (3)
C2—C3	1.385 (4)	C15—O5	1.245 (3)
C2—H2	0.9300	C15—N7	1.305 (3)
C3—C4	1.377 (4)	C15—H15	0.9300
C3—N1	1.452 (3)	C16—N7	1.440 (4)
C4—C5	1.377 (4)	C16—H16A	0.9600
C4—H4	0.9300	C16—H16B	0.9600
C5—C6	1.400 (3)	C16—H16C	0.9600
C5—H5	0.9300	C17—N7	1.453 (4)
C6—N2	1.398 (3)	C17—H17A	0.9600
C7—N3	1.162 (3)	C17—H17B	0.9600
C7—N2	1.301 (3)	C17—H17C	0.9600
C8—C9	1.375 (4)	Co1—O5	1.9807 (19)
C8—C13	1.397 (3)	Co1—N3	1.994 (2)
C8—H8	0.9300	Co1—N5	2.198 (2)
C9—C10	1.383 (4)	Co1—N6^i^	1.971 (2)
C9—H9	0.9300	Co1—N2^i^	2.2896 (19)
C10—C11	1.375 (4)	N1—O2	1.220 (4)
C10—N4	1.461 (3)	N1—O1	1.222 (4)
C11—C12	1.377 (4)	N2—Co1^ii^	2.2896 (19)
C11—H11	0.9300	N4—O3	1.226 (5)
C12—C13	1.400 (4)	N4—O4	1.231 (4)
C12—H12	0.9300	N6—Co1^ii^	1.971 (2)
			
C2—C1—C6	120.5 (2)	N7—C16—H16A	109.5
C2—C1—H1	119.7	N7—C16—H16B	109.5
C6—C1—H1	119.7	H16A—C16—H16B	109.5
C1—C2—C3	119.5 (2)	N7—C16—H16C	109.5
C1—C2—H2	120.3	H16A—C16—H16C	109.5
C3—C2—H2	120.3	H16B—C16—H16C	109.5
C4—C3—C2	121.3 (2)	N7—C17—H17A	109.5
C4—C3—N1	119.4 (3)	N7—C17—H17B	109.5
C2—C3—N1	119.2 (3)	H17A—C17—H17B	109.5
C3—C4—C5	119.3 (3)	N7—C17—H17C	109.5
C3—C4—H4	120.3	H17A—C17—H17C	109.5
C5—C4—H4	120.3	H17B—C17—H17C	109.5
C4—C5—C6	120.9 (2)	N6^i^—Co1—O5	114.40 (10)
C4—C5—H5	119.5	N6^i^—Co1—N3	131.52 (10)
C6—C5—H5	119.5	O5—Co1—N3	114.07 (9)
N2—C6—C5	122.8 (2)	N6^i^—Co1—N5	90.32 (8)
N2—C6—C1	118.7 (2)	O5—Co1—N5	92.72 (8)
C5—C6—C1	118.4 (2)	N3—Co1—N5	88.66 (8)
N3—C7—N2	175.0 (2)	N6^i^—Co1—N2^i^	85.75 (8)
C9—C8—C13	120.4 (3)	O5—Co1—N2^i^	93.78 (8)
C9—C8—H8	119.8	N3—Co1—N2^i^	89.94 (8)
C13—C8—H8	119.8	N5—Co1—N2^i^	173.35 (8)
C8—C9—C10	119.3 (3)	O2—N1—O1	122.8 (3)
C8—C9—H9	120.4	O2—N1—C3	118.1 (3)
C10—C9—H9	120.4	O1—N1—C3	119.1 (3)
C11—C10—C9	121.7 (2)	C7—N2—C6	117.25 (19)
C11—C10—N4	119.6 (3)	C7—N2—Co1^ii^	114.63 (15)
C9—C10—N4	118.7 (3)	C6—N2—Co1^ii^	123.69 (13)
C10—C11—C12	119.1 (3)	C7—N3—Co1	159.71 (19)
C10—C11—H11	120.4	O3—N4—O4	123.6 (3)
C12—C11—H11	120.4	O3—N4—C10	118.2 (3)
C11—C12—C13	120.6 (3)	O4—N4—C10	118.2 (3)
C11—C12—H12	119.7	C14—N5—C13	117.8 (2)
C13—C12—H12	119.7	C14—N5—Co1	115.24 (16)
C8—C13—C12	118.9 (2)	C13—N5—Co1	123.95 (14)
C8—C13—N5	118.6 (2)	C14—N6—Co1^ii^	164.6 (2)
C12—C13—N5	122.5 (2)	C15—N7—C16	121.6 (3)
N6—C14—N5	173.8 (3)	C15—N7—C17	121.1 (3)
O5—C15—N7	123.9 (3)	C16—N7—C17	117.3 (3)
O5—C15—H15	118.0	C15—O5—Co1	128.61 (18)
N7—C15—H15	118.0		
			
C6—C1—C2—C3	0.2 (4)	C1—C6—N2—Co1^ii^	37.3 (3)
C1—C2—C3—C4	−0.9 (4)	N6^i^—Co1—N3—C7	−104.7 (6)
C1—C2—C3—N1	179.0 (2)	O5—Co1—N3—C7	76.9 (6)
C2—C3—C4—C5	0.8 (4)	N5—Co1—N3—C7	−15.5 (6)
N1—C3—C4—C5	−179.1 (3)	N2^i^—Co1—N3—C7	171.0 (6)
C3—C4—C5—C6	0.0 (4)	C11—C10—N4—O3	−5.4 (4)
C4—C5—C6—N2	178.2 (2)	C9—C10—N4—O3	175.4 (3)
C4—C5—C6—C1	−0.7 (4)	C11—C10—N4—O4	175.3 (3)
C2—C1—C6—N2	−178.4 (2)	C9—C10—N4—O4	−3.9 (4)
C2—C1—C6—C5	0.7 (4)	C8—C13—N5—C14	157.8 (2)
C13—C8—C9—C10	−0.9 (4)	C12—C13—N5—C14	−23.2 (4)
C8—C9—C10—C11	1.9 (4)	C8—C13—N5—Co1	−42.7 (3)
C8—C9—C10—N4	−178.9 (3)	C12—C13—N5—Co1	136.2 (2)
C9—C10—C11—C12	−1.4 (4)	N6^i^—Co1—N5—C14	133.7 (2)
N4—C10—C11—C12	179.4 (3)	O5—Co1—N5—C14	−111.83 (19)
C10—C11—C12—C13	0.0 (4)	N3—Co1—N5—C14	2.21 (19)
C9—C8—C13—C12	−0.5 (4)	N6^i^—Co1—N5—C13	−26.2 (2)
C9—C8—C13—N5	178.5 (2)	O5—Co1—N5—C13	88.3 (2)
C11—C12—C13—C8	0.9 (4)	N3—Co1—N5—C13	−157.7 (2)
C11—C12—C13—N5	−178.0 (2)	O5—C15—N7—C16	0.4 (4)
C4—C3—N1—O2	178.9 (3)	O5—C15—N7—C17	−178.6 (3)
C2—C3—N1—O2	−1.0 (5)	N7—C15—O5—Co1	172.1 (2)
C4—C3—N1—O1	−0.1 (4)	N6^i^—Co1—O5—C15	−63.6 (3)
C2—C3—N1—O1	−180.0 (3)	N3—Co1—O5—C15	115.0 (2)
C5—C6—N2—C7	13.3 (4)	N5—Co1—O5—C15	−155.2 (2)
C1—C6—N2—C7	−167.7 (2)	N2^i^—Co1—O5—C15	23.4 (3)
C5—C6—N2—Co1^ii^	−141.7 (2)		

Symmetry codes: (i) *x*, −*y*+1/2, *z*+1/2; (ii) *x*, −*y*+1/2, *z*−1/2.
